# Frequency of incidental ocular findings during pre-employment screening at a tertiary care Eye hospital

**DOI:** 10.12669/pjms.37.3.3177

**Published:** 2021

**Authors:** Summaya Khan, Aisha Rafique, Omar Zafar

**Affiliations:** 1 Dr. Summaya Khan, FCPS., Armed Forces Institute of Ophthalmology, Rawalpindi, Pakistan; 2 Dr. Aisha Rafique, MBBS., Armed Forces Institute of Ophthalmology, Rawalpindi, Pakistan; 3 Dr. Omar Zafar, MCPS, FCPS., Armed Forces Institute of Ophthalmology, Rawalpindi, Pakistan

**Keywords:** Incidental findings, Pre-employment ocular screening, Routine eye examination

## Abstract

**Objective:**

To highlight the prevalence of incidentally discovered ocular findings harvested amongst candidates of different age groups presented for pre-employment screening in a tertiary care eye hospital.

**Methods:**

This Cross sectional prospective study was conducted in Armed Forces Institute of Ophthalmology, Rawalpindi, from Jun 2018 to Dec 2019. Data was collected using non-probability consecutive sampling technique. All candidates who appeared for medical fitness examination were included. Candidates belonged to various regions of Pakistan. Complete ophthalmic checkup including visual acuity, best corrected visual acuity, anterior and posterior segment examination was performed. The data analysis was done by IBM SPSS 2.0 software.

**Results:**

One thousand and five hundred (1500) candidates underwent ophthalmic medical fitness examination during Jun 2018 to Dec 2019, out of these 86% (1290) were males and 14% (210) were females. Mean age of the candidates was 23.14 ± 5.66 years. The most common incidental ocular findings were amblyopia 24.6% (369), strabismus 10% (150), cataract 7.3% (110), macular scar 6.5% (100) and colour vision deficiencies 5.5% (82).

**Conclusion:**

The study demonstrates that out of total patients, 77% (1095) were found to be asymptomatic and 23% (405) were symptomatic. The study provides frequency for prevailing diseases and can help in improvement of eye care screening.

## INTRODUCTION

The incidence and prevalence of various ophthalmic diseases in a community varies with the different socio-environmental factors. The prevalence of commonly occurring diseases are well known, at least in developed countries where public health data are collected regularly and pertinently.^1^ Ample valuable information has emerged from the population-based epidemiologic studies of eye disease undertaken over the past decade. Most significant finding is the negative impact that even a relatively moderate decrease in visual acuity (<6/12) imposes on individual’s quality of life and his ability to endeavor accomplishments.^2^ These studies have also stressed the exponential increase in ocular disorders and vision loss with advancing age.^3^ This research has embarked an emphasis for the need of regular vision checkups and help practitioners to consider who is at risk among their patients in order to timely diagnose ongoing pathological process.

In some cases, a higher prevalence of a particular disease in a population can encourage healthcare delivering administration to design an adaptable screening program aiding to implement a new treatment strategy. It aims towards decreasing morbidity and shrink disease-associated costs. In all these cases, education of high risk patients or those having a familial history of ocular disease may prompt early detection, limiting the costs necessary to treat advance stages of disease.^4^ Besides all conditions mentioned above, the prime manifesto is prevention of visual loss, the privileged sense of all living beings.

The key constituent of improving eye health is need for regular eye checkups, as many eye diseases can exist and progress relentlessly without the individual being aware of the problem until much vision is lost.^5^ Glaucoma is a classic example of such kind. It can be well diagnosed in time by prompt screening and follow up.^6^

In our study, incidental ocular findings in individuals who were otherwise healthy helped in the recognition that reduced vision can have a serious impact in terms of morbidity. It lays an emphasis on psycho-social issues when there was rejection of young individual right at the initial stages of pre-employment screening. It also serves as a medium of public health message regarding decreased vision from various etiological factors. Our study provides information to protect eyes from damaging effects of UV rays, pre emptive glaucoma screening and need of paying significant attention any visual symptom.

## METHODS

This study was conducted over a period of one and a half year from June 2018 till December 2019 in Armed Forces Institute of Ophthalmology after taking approval from hospital ethical review committee (ERC-250/AFIO). In this cross-sectional study, a total of 1500 participants were included using non probability consecutive sampling technique. Sample size was calculated using WHO sample size calculator, keeping confidence interval of 95% and 5% error.

The study included all candidates appearing for pre-employment ocular examination. Individuals with history of any ocular surgery or neurosurgery, trauma, systemic medication that has ocular side effects like anti-tuberculosis treatment or central nervous system drugs and previous history of intra ocular injections were excluded from the study. Patient demographic data such as age, gender and place of referral were noted. Medical history including diabetes mellitus, hypertension, and other systemic diseases was documented. Informed consent was taken and they were briefly described about examination and purpose of study. Ophthalmic examination included unaided vision and best corrected visual acuity. High refractive error and amblyopia was ruled out during refraction. Individuals with -6.00 to + 6.00 D refractive error were considered to have high myopia or high hypermetropia respectively.^7^ High astigmatism was considered with error of 4 D.^8^ Amblyopia was defined as unilateral or bilateral decrease in best corrected visual caused by form vision deprivation or faulty binocular interaction in absence of identifiable organic ocular pathology.^9^ Extraocular movements and adnexal / orbital examination was performed to evaluate proptosis which was further confirmed by using Hertels exo-ophthalmometer. Detailed anterior and posterior segment examination was performed via slit lamp (Haag-Streit BM 900 Unit LED Slit lamp). Intra ocular pressure measured (IOP) with air puff tonometer (Nidek NT-530/510). >21 mm of Hg was confirmed with Goldman applanation tonometer. It was further supplemented with central corneal thickness measurement, optic disc evaluation, visual fields, optical coherence tomography of retinal nerve fiber layer to diagnose glaucoma or glaucoma suspects.

Detailed funduscopy using 90 D lens and by indirect ophthalmoscope for peripheral retinal evaluation was performed. Pertinent investigations were performed to confirm suspected relevant conditions. Color vision deficiency was checked by using pseudo-isochromatic Ishihara color plates which were shown to patient monocularly at a distance of 75 cm in well illuminated ambience. Colour vision was checked after full correction of refractive error. Plates were presented at least for 6 to 12 seconds and candidates were asked to identify numbers or patterns of test plates.^10^,^11^

Data was entered and analyzed in SPSS version 2.0. Descriptive statistics were used to calculate mean and standard deviation of age. Percentage and frequency was calculated for prevalent ocular findings.

## RESULTS

One thousand and five hundred (1500) candidates underwent ophthalmic medical fitness examination during Jun 2018 to Dec 2019, out of these 1290 (86%) were male and 210 (14%) were female. Mean age of the candidates was 23.14 ± 5.66 years (Table-I). The most common incidental ocular findings were amblyopia 24.6% (369), strabismus 10% (150), cataract 7.5% (110), macular scar 6.5% (100) and colour vision deficiencies 5.5% (82) (Table-II). Out of total patients, 73% (1095) were found to be asymptomatic and 27% (405) were symptomatic.

**Table-I:** Age and Gender Distribution.

Age Distribution		

Age Group – Years	Number of Cases	Percentage (%)
10 – 20	615	41
21 – 30	517	34.5
31 – 40	270	18
41 - 50	98	6.5

*Gender Distribution*		

Males	1290	86
Females	210	14
Total	1500	100

**Table-II:** Incidence of Various Eye Diseases.

Serial No	Diagnosis	No of Cases	Percentage (%)
1	Amblyopia	369	24.6
2	Strabismus	150	10
3	Cataract	110	7.5
4	Macular Scar	100	6.5
5	Colour Vision Defect	82	5.5
6	Corneal Opacity	75	5.0
7	Retinal Degeneration	66	4.4
8	Corneal Dystrophy	60	4.0
9	Trachoma	50	3.3
10	High Refractive Error	50	3.3
11	Pterygium	50	3.3
12	Allergic Conjunctivitis	50	3.3
13	Keratoconus	45	3.0
14	Presbyopia	42	2.8
15	Retinochoroidal Coloboma	25	1.7
16	Uveitis	25	1.7
17	Glaucoma	25	1.7
18	Ptosis	25	1.7
19	Proptosis	25	1.7
20	Nystagmus	25	1.7
21	Refractive Surgery	17	1.1
22	Optic Disc Drusen	14	0.93
23	Posterior Staphyloma	7	0.46
24	Retinal Detachment	5	0.33
25	Contact Lens	4	0.26
27	Episcleritis / Scleritis	4	0.3
Total		1500	100

## DISCUSSION

Vision is extremely treasured sense as we remark maximum imprints of our world by means of sight. It is the utmost researched sense than any other sensory modality. Intention for this is seemingly simple: because vision is most significant and intricate sense.^12^ Those who are agonized from visual impairment are more prone to be unemployed, socially isolated and dependent as age passes by. In our country with financial constrain, very few people have access to or have information regarding low vision rehabilitation centers. Girls suffering from syndromic low vision are not even allowed to be sent to visual rehabilitation schools owing to social stigma in certain regions of Pakistan. It supplements profound psychological and social implications.^13^

Our study reveals that regular comprehensive eye checkups are the best way to detect pathology for asymptomatic and symptomatic patients. It deems to devise timely preventive and curative treatment strategies. Several disorders like cataract, glaucoma amblyopia and macular degeneration are often asymptomatic or symptoms are not well appreciated by the patient at time of initial presentation. Ophthalmologists acknowledge that aforementioned disease processes are preventable and curable if identified at an early phase in disease progression.^14^

There were significant number of patient with amblyopia which was shockingly more than prevalence in other studies on Eastern population.^15^ Most of them were well above the age of neuroplasticity and has never undergone occlusion therapy or any other treatment modality for amblyopia. Neuroplasticity is capability of brain to reorganize its neuronal pathways. However, there is a certain age limit beyond which this remapping of neuronal mechanism is not possible. The reason for increased prevalence of amblyopia noted in our study (24.6%) may be attributed to higher age groups participating in our study which is comparable to results of study undertaken by Hamid S et al which was 22.7%.^16^ These untreated patients can have significant visual impairment persisting throughout their lives.

Cataract is a leading cause of reversible blindness.^17^ In our study cataract was also recognized as a significant incidental finding. Most of the candidates in our study were asymptomatic. It included blue dot, sutural, posterior polar and lamellar cataract affecting mild to moderate vision loss of < 6/12. Over the time these patches of lenticular opacity become bigger and blurry eventually leading to marked visual loss.^18^

Keratoconus prevalence in our population is comparable to results of the international data. Main assosciation is vernal kerato-conjunctivitis in all the individuals. Keratoconus is considered as debilitating visual pathology and considered as disease of a young population. Steepening and thinning of cornea in keratoconus is associated with sight threatening complications. Treatment ranges from spectacles to expensive advance refractive procedures.^19^

There were 50 patients out of 1500 who had pterygium and all of them were males. Some of them deployed or were residents at higher altitudes leading to implication of increased solar exposure. Patient were educated regarding the disease and treatment which entails surgical removal if it obscures visual axis.

Prevalence of high refractive error was 3.3 percent. It is comparable to other published studies.^20^ Myopia was found as most common high refractive error (64.5%), high hypermetropia (21%) and high astigmatism (14.5%). Astigmatism was found to be most common refractive error (68.6%). High myopia is associated with retinal degenerations and premature posterior vitreous detachment leading to symptomatic break in retina.^21^ These entities demand urgent surgical repair with ensured availability of advance instrumentation and expertise which if not timely undertaken leads to total blindness.^22^ Prevalence of corneal opacities varies with one region of world to another. Main causes identified were trauma, road traffic accident, trachoma and miscellaneous infectious etiology.^23^

Strabismus is associated with short term (diplopia, headache, confusion) and long term effects (abnormal or absent binocular vision, unacceptable cosmesis).^24^ Frequency of strabismus in our study group is comparable to national data. Most common strabismus was eso-deviation under less than 20 years of age group (1.7%). According to different surveys, eso-deviation is approximately three times common in children and younger age groups. However, exo-deviation is more common in age group greater than 20 years (3.3%).^25^ Majority of cases were manifest strabismus in our study.

One entity worth mentioning is raised frequency of color blindness found among individual. Out of these, 94.52% were absolutely unaware of their anomalous visual status. It is comparable to other research study undertaken in southern Punjab province of Pakistan which showed prevalence of 93.54%.^26^ Although it is non pathological, non-progressive and incurable, it poses major risk in rejection for specified jobs like aviation, defense, railway employment in younger population striving and pursuing for their livelihood.

Allergic conjunctivitis is an all-encompassing term that comprises of seasonal allergic conjunctivitis, worsening with seasonal variations, vernal kerato-conjunctivitis which has established connotation in development of keratoconus and atopic kerato-conjunctivitis which exacerbates on exposure to specific recognized antigen.^27^ In our study, 3.3 percent had allergic conjunctivitis. Most had mild symptoms.

One of the significant finding is presence of atrophic macular scar (6.5%), the prevalence is comparable to internationally published study by Machado RAF et al which was 6.38%.^28^ 98% were healed associated with trauma in childhood and toxoplasmosis. Atrophic scars present in foveal region severely compromised central visual field and almost all patient with foveal scar having central visual field defects.

There were 73% of asymptomatic and 27% symptomatic patients. These asymptomatic patients were incidentally found thorough ophthalmic examination harboring pathology or at least having risk of developing pathology warranting necessity of treatment and follow up. Our study, which was conducted in a particular proportion of population has emphasized on the fact that percentage of incidental findings observed during pre-employment screening if extrapolated to total no of patients visiting for routine eye examination, the resultant number of affected individuals would be substantially high.

### Limitation of the study:

Our study was limited as only those patients who had ocular findings were included. So systemic associations couldn’t be found. However, these patients were further referred to specialized clinics for treatment and visual rehabilitation.

## CONCLUSION

Our study has given us insight that ocular examination is much more than mere refraction and prescription of glasses as believed by our general population. It has provided a scaffold in identification of ocular diseases. It is demonstrated to expedite incidental findings as harbinger for necessary referral and timely intervention to prevent vision loss.

### Author’s Contribution:

**SK:** Conceived, data collection and is responsible for integrity of the study.

**AR:** Data collection, statistical analysis and manuscript writing.

**OZ:** Review and final approval.
